# Supplementary material for: Identification of Cold Tolerance Transcriptional Regulatory Genes in Seedlings of Medicago sativa L. and Medicago falcata L
Source: Int J Mol Sci. 2024 Sep 26;25(19):10345. doi: 10.3390/ijms251910345 (PMC11476818; doi:10.3390/ijms251910345)
Supplement: Supplementary file 1 [file ijms-25-10345-s001.zip › Supplementary Figures S1-S3.pdf]

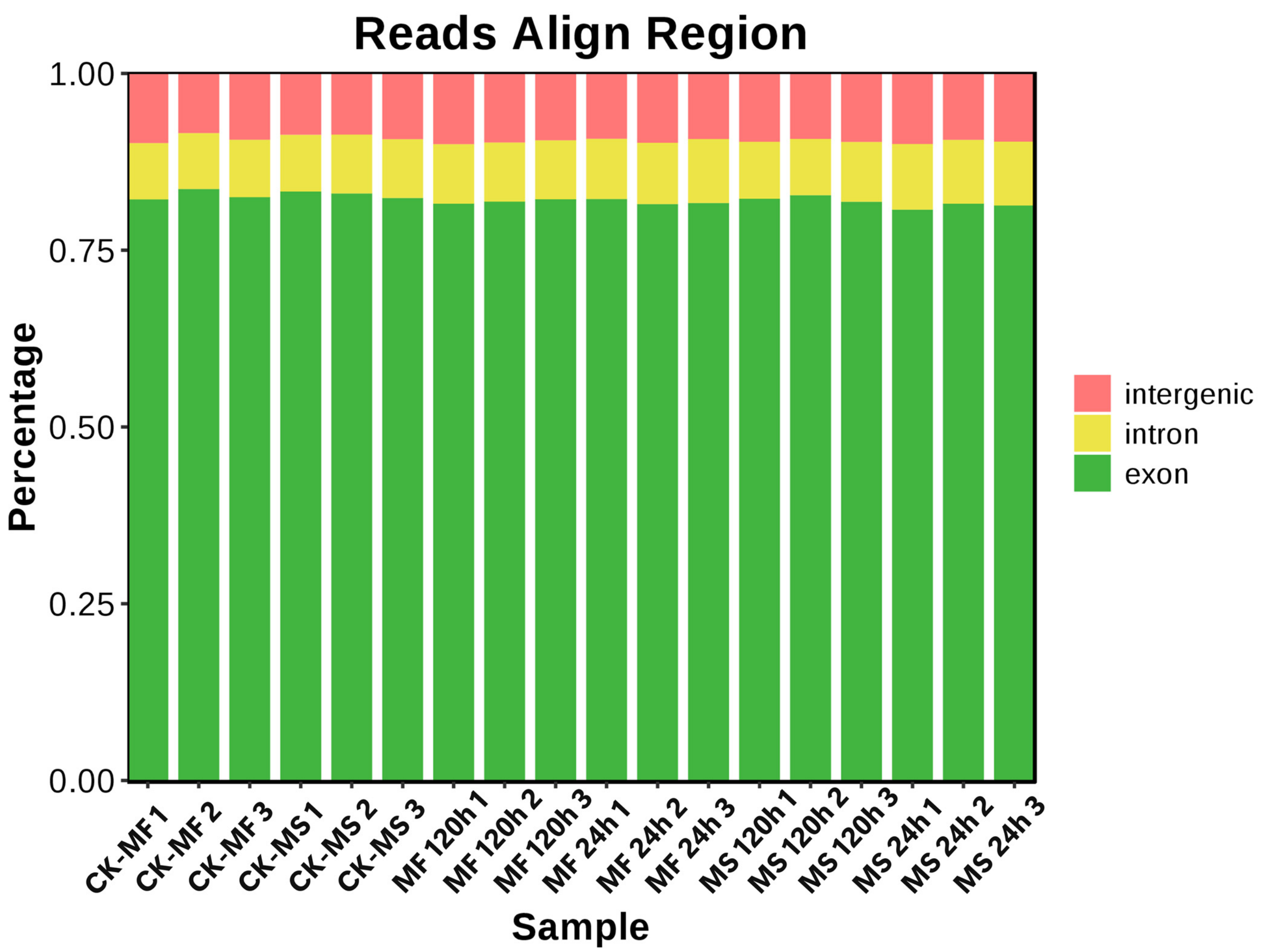

Figure S1. Comparison of reference regions. The abscissa represents the sample, and the ordinate represents the percentage of reads in the reference genome.

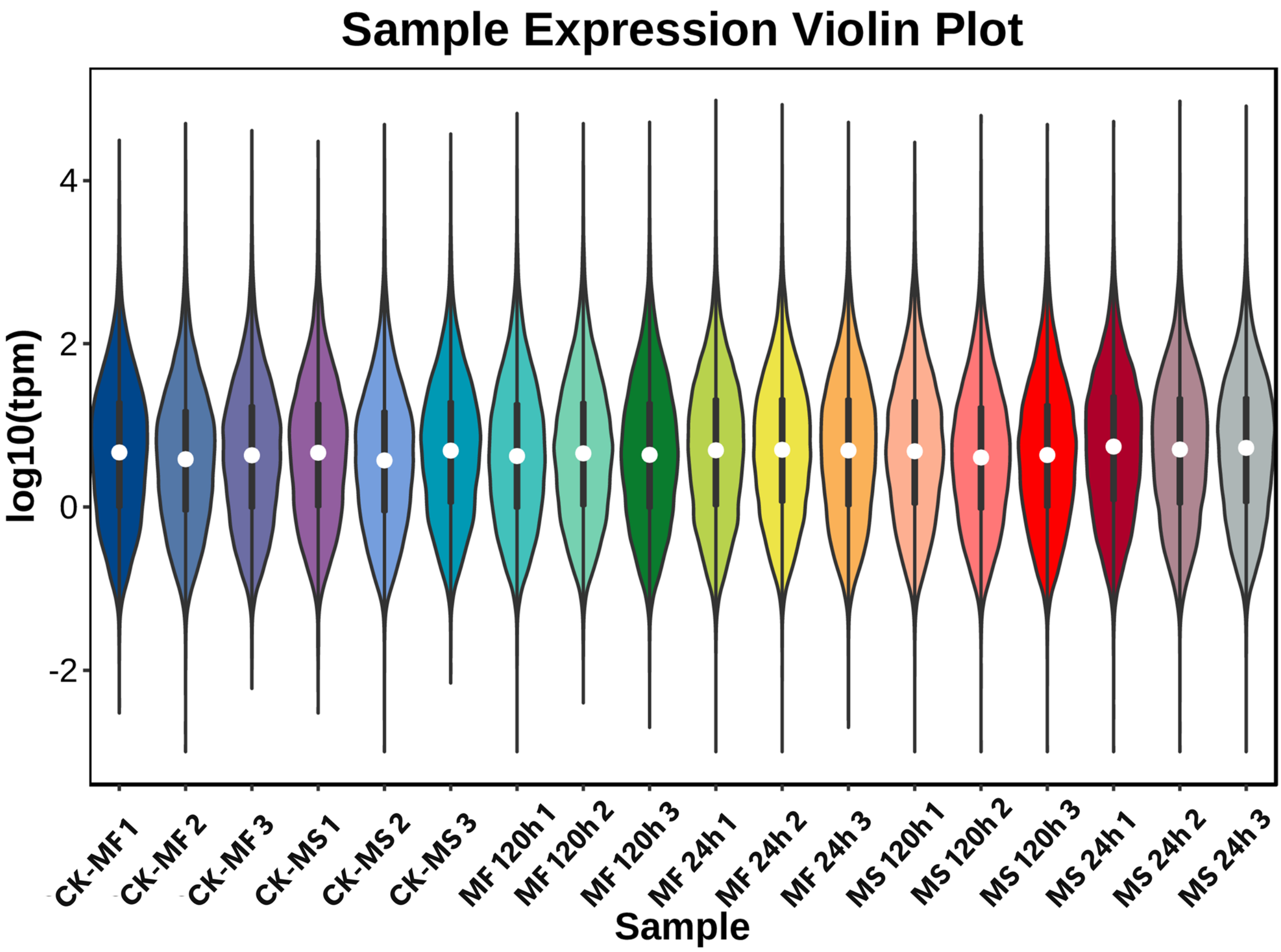

Figure S2. Violin diagram of gene expression. The white dot represents the median; The black rectangle is the range from the lower quarttile to the upper quartile; The length represents the degree of dispersion and symmetry of the non-anomalous data, and the black line represents the range from the minimum non-outlier to the maximum non-anomalous value. The vertical length of the graph represents the degree of data diffusion, and the horizontal length represents the amount of data distribution at a certain ordinate position.

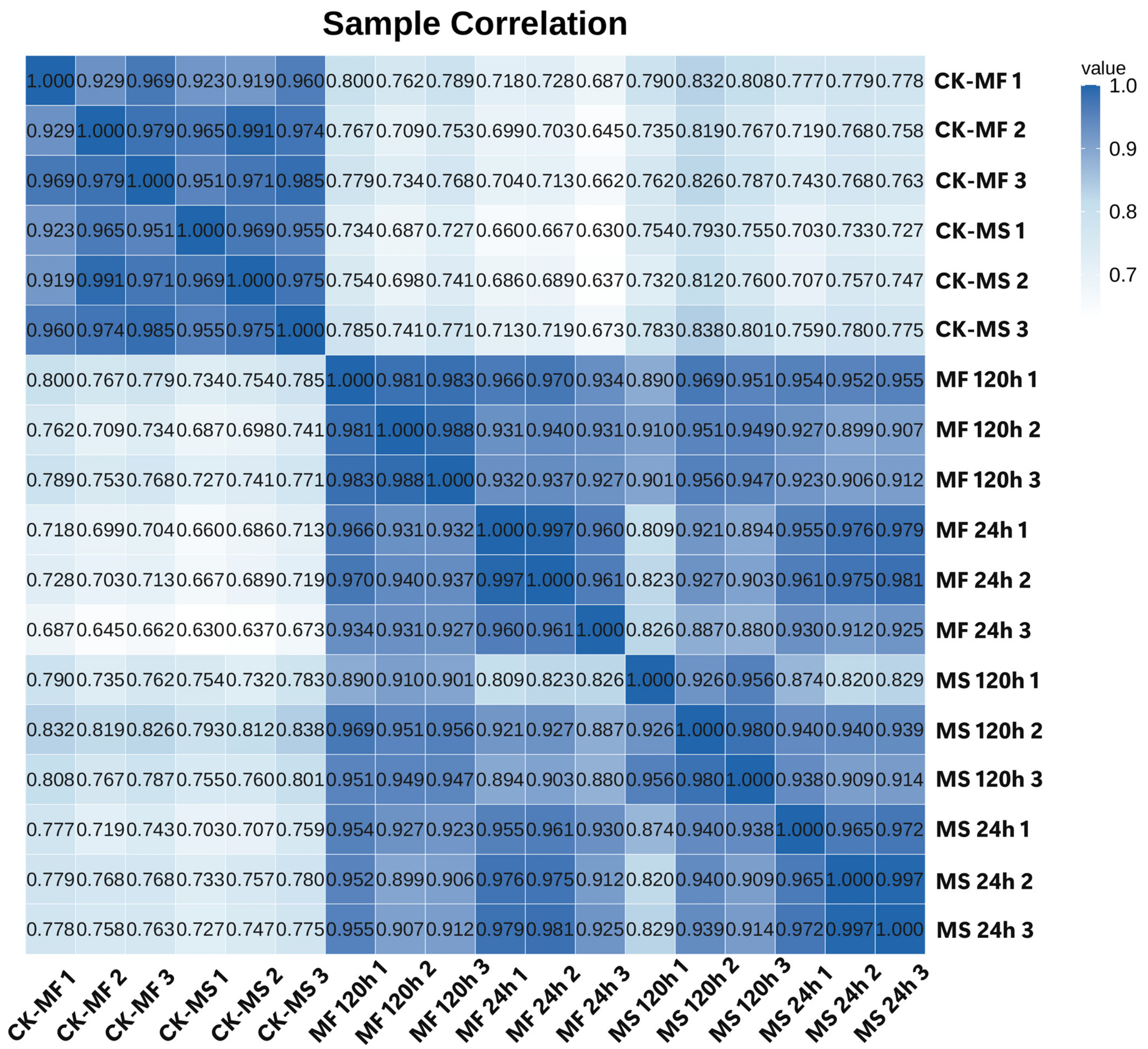

Figure S3. Expression correlation heatmap analysis. In the figure, the abscissa and ordinate are for each sample, and the color shade indicates the size of the correlation coefficient between the two samples. The closer to blue is the greater the correlation, and the closer to white the is, the smaller the correlation.
